# Diabetic Neuropathy: New Insights to Early Diagnosis and Treatments

**DOI:** 10.1155/2018/5378439

**Published:** 2018-12-10

**Authors:** Mark Yorek, Rayaz A. Malik, Nigel A. Calcutt, Aaron Vinik, Soroku Yagihashi

**Affiliations:** ^1^University of Iowa and Iowa City VA Healthcare System, Iowa City, USA; ^2^Weill Cornell Medicine-Qatar, Doha, Qatar; ^3^University of Manchester, Manchester, UK; ^4^University of California, San Diego, USA; ^5^Eastern Virginia Medical School, Norfolk, USA; ^6^Hirosaki University Graduate School of Medicine, Hirosaki, Japan

Diabetic peripheral neuropathy (DPN) is a complex disorder with multiple etiologies that affects about 50% of those with diabetes, and about 30% have painful diabetic neuropathy. Neuropathy can also develop in subjects and animal models with prediabetes/insulin resistance, but without overt hyperglycemia. At this time, there is no known treatment and good glycemic control slows progression of DPN in type 1 diabetes, but not type 2 diabetes [1]. The annual cost for healthcare in patients with DPN in the United States is staggering with estimates exceeding $15.0 billion [2]. Given the increasing incidence of both type 1 and type 2 diabetes and obesity and the impact DPN has on the quality of life of patients and their families and overall healthcare costs, a strategy for the early diagnosis and discovery of an effective treatment is needed.

The early diagnosis and staging of the severity of diabetic neuropathy using patient questionnaires, determining thermal and mechanical sensitivities, and examining nerve conduction velocities require standardization. Whilst recent studies have established good diagnostic utility and reproducibility for sensory nerve evaluation in the skin and cornea, there has been limited application of these techniques in the clinic [3]. For patients with painful diabetic neuropathy, underdiagnosis and adequate control of symptoms remain challenging.

Animal studies have provided considerable insight to the etiology and treatment of diabetic neuropathy. However, there has been a uniform failure to translate these findings to an FDA-approved therapy. Many factors may account for these poor outcomes and need to be addressed at multiple levels. Preclinical studies need to use more predictive/translational endpoints and combination therapies, to identify treatment that at a minimum will slow progression or even repair nerve damage caused by prediabetes/diabetes.

These challenges in DPN lead us to pursue a special issue incorporating the latest clinical and preclinical studies on the diagnosis and treatment of diabetic neuropathy.

F. Picconi et al. assessed whether retinal neurodegeneration has a predictive value in the development of peripheral motor as opposed to sensory unit loss and complimented the work assessing the utility of corneal nerve loss acting as a surrogate marker for DPN [4]. They show that motor unit loss and neuroretinal dysfunction already exist in patients with type 1 diabetes without classical DPN. They concluded that the neuroretina is a potential “window” onto the early neurogenic process in diabetes. These and other studies (see below) suggest that cornea and retinal neurodegeneration may allow early diagnosis of DPN.

X. Jia et al. investigated whether in vivo corneal confocal microscopy could detect improvement of corneal nerve parameters following improved glycemic control in patients with type 2 diabetes. 32 patients with DPN and 12 age-matched control subjects underwent nerve conduction studies and assessment of corneal nerve morphometry at baseline and after approximately one year. At follow-up, 1/2 of the diabetic subjects had improved HbA1c whilst the other 1/2 continued to have elevated HbA1c. In the subjects with improved glycemic control, corneal nerve fiber density and corneal nerve fiber length increased significantly compared to baseline, whilst those with poor HbA1c showed a significant reduction in sural sensory nerve conduction velocity, corneal nerve fiber density, and corneal nerve fiber length. The authors concluded that corneal nerve fiber repair can be detected when glycemic control improves and that in vivo corneal confocal microscopy could be a sensitive method to assess nerve repair in future longitudinal or interventional studies of DPN.

Sensory nerves in the skin can also be examined as a potential marker for nerve repair in DPN [5]. Saudek et al. examined whether there was a change in the expression of mRNA levels of neurotrophic growth factors and nerve regeneration in the skin 28 months after renal and pancreas transplantation and normalization of glycemia. Transplantation upregulated mRNA levels of multiple neurotrophic growth factors in the skin but was not accompanied by an improvement in skin nerve fiber density or electrophysiological indices of DPN.

R. B. Jansen et al. assessed whether markers of inflammation could be detected in acute Charcot foot compared to the healthy foot. Five subjects with acute Charcot foot were found to have an increased venous-arterial flux of interleukin-6 (IL-6) in the acute diabetic Charcot foot compared to the healthy foot. They also found an increased level of IL-6 and advanced glycation end products in the acute Charcot foot after externally cooling the feet, with no difference in the flux for other markers of inflammation.

B. L. M. Van Eetvelde et al. assessed the influence of clinically diagnosed DPN on respiratory muscle strength in patients with type 2 diabetes. They found that DPN affects respiratory muscle strength in type 2 diabetes patients and concluded that the assessment of respiratory muscle weakness could be of added value in the early screening for DPN in patients with type 2 diabetes.

T. Meldgaard et al. reviewed the diabetes-induced structural remodeling, biochemical dysfunction, immune-mediated alterations, and inflammatory properties of the enteric nervous system and associated structures. They discussed the various patient-reported outcome measures and objective modalities for evaluating dysmotility and outlined the clinical management of diabetic enteropathy. They advocated prompt investigation and intervention, to improve the quality of life for patients with this condition.

M. Bulc et al. explored novel underlying mechanisms of diabetic enteropathy by using double immunofluorescent labelling of substance P, calcitonin gene-related peptide, and leu5-enkephalin on enteric stomach neurons of a porcine model of chemically induced diabetes. The percentage of neurons immunoreactive to substance P and calcitonin gene-related peptide in the myenteric plexus of the antrum, corpus, and pylorus as well as in the submucosal plexus of the corpus was increased. Whilst the number of leu5-enkephalin-like immunoreactivity neurons was increased in the myenteric plexus of the antrum and the submucosal plexus of the corpus, there was a decrease in the myenteric plexus of the corpus and pylorus. The authors concluded that these substances may participate in the development of gastric complications and that future research on the role of these peptides may result in the development of effective pharmacotherapy of gastrointestinal disorders.

In an animal model of type 2 diabetes that combined high-fat feeding and low-dose streptozotocin treatment of Sprague-Dawley rats, E. P. Davidson et al. reported that dietary salsalate in combination with lower amounts of menhaden oil provided greater benefit in diabetes-induced vascular and neural impairment than menhaden oil alone, stressing the importance of combination therapy for the treatment of DPN.

Overall, many of the articles appearing in this special issue stress the importance of early detection for DPN and the need for a successful treatment.
